# The recombinase activating genes: architects of immune diversity during lymphocyte development

**DOI:** 10.3389/fimmu.2023.1210818

**Published:** 2023-07-11

**Authors:** Merijn Braams, Karin Pike-Overzet, Frank J. T. Staal

**Affiliations:** ^1^Department of Immunology, Leiden University Medical Centre, Leiden, Netherlands; ^2^Novo Nordisk Foundation Centre for Stem Cell Medicine (reNEW), Leiden University Medical Centre, Leiden, Netherlands; ^3^Department of Paediatrics, Leiden University Medical Centre, Leiden, Netherlands

**Keywords:** BCR - B cell receptor, TCR - T cell receptor, rearrangements of immunoglobulin and T cell receptor genes, gene therapy (GT), thymus, bone marrow, Recombination activating genes

## Abstract

The mature lymphocyte population of a healthy individual has the remarkable ability to recognise an immense variety of antigens. Instead of encoding a unique gene for each potential antigen receptor, evolution has used gene rearrangements, also known as variable, diversity, and joining gene segment (V(D)J) recombination. This process is critical for lymphocyte development and relies on recombination-activating genes-1 (RAG1) and RAG2, here collectively referred to as RAG. RAG serves as powerful genome editing tools for lymphocytes and is strictly regulated to prevent dysregulation. However, in the case of dysregulation, RAG has been implicated in cases of cancer, autoimmunity and severe combined immunodeficiency (SCID). This review examines functional protein domains and motifs of RAG, describes advances in our understanding of the function and (dys)regulation of RAG, discuss new therapeutic options, such as gene therapy, for RAG deficiencies, and explore *in vitro* and *in vivo* methods for determining RAG activity and target specificity.

## 1 Introduction

Throughout an individual’s lifetime, the immune system is exposed to numerous foreign antigens that must be promptly cleared before they can inflict substantial damage. While the innate immune response is often capable of handling such intruders without requiring additional support, there are instances where the adaptive immune response must coordinate a more elaborate defence strategy. To accomplish this, T and B lymphocytes from the adaptive immune response must be equipped to launch a response against any possible foreign agent that may invade the body.

Rather than encoding separate genes for each possible antigen receptor, the immune system has “invented” a mechanism of DNA rearrangement, known as V(D)J recombination. This mechanism allows for the antigen recognition gene segments of lymphocytes to be modified, creating one of the largest biological information banks in the world, capable of generating a vast repertoire of trillions of possible combinations (1). At the heart of this process lie RAG1 and RAG2, collectively referred to as RAG.

**Figure 1:**
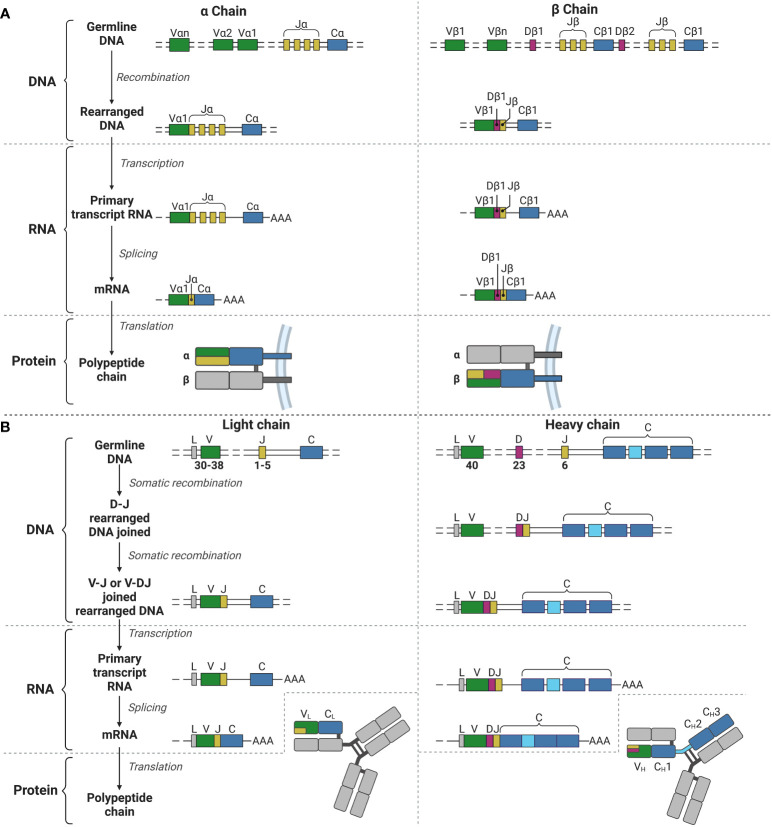
V(D)J recombination for the TCR α and β chains and the BCR light and heavy chains. **(A)** V(D)J recombination for the α (left) and β (right) chains for the TCR in T lymphocyte development. **(B)** V(D)J recombination for the light (left) and heavy (right) chains for the BCR (and immunoglobulins) in B lymphocyte development. V, Variable; J, joining; C, constant; D, diversity; L, leader; AAA, poly-A-tail. Created with BioRender.com.

The RAG complex is a unique endonuclease that is responsible for inducing intentional DNA double-strand breaks (DSBs). RAG does so specifically around certain nonamer and heptamer sequences referred to as recombination signal sequences (RSSs) that flank the V, D and J segments in the genome (1, 2). By excising different variants of V(D)J segments, RAG (along with DNA repair mechanisms) creates coding joints that code for specific B-cell receptor (BCR) or T-cell receptor (TCR) gene segments. Thereby, generating clonal diversity (see **Figure 1**) from a relatively short piece of DNA when taking into perspective the number of different receptor possibilities (1, 2). As a result of its critical role in the V(D)J recombination process, RAG is indispensable for lymphocyte development. Loss-of-function mutations in RAG can completely block lymphocyte development at an early stage, leading to SCID (3, 4). On the other hand, dysregulation of RAG has been associated with autoimmunity and RAG-mediated oncogenic fusion genes that promote blood-borne cancer formations such as acute lymphoblastic leukaemia (ALL) (5). Therefore, the regulation of RAG is of the utmost importance to prevent dysregulation and adverse outcomes.

## 2 RAG protein and the recombination process from germline to coding joint

### 2.1 RAG protein structure and functional domains

**Figure 2:**
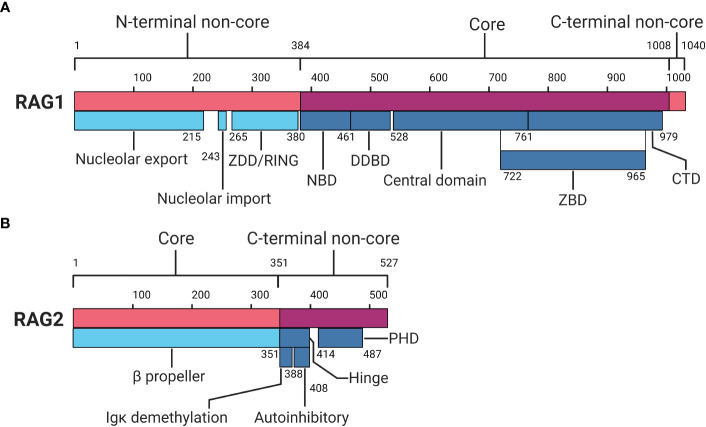
Protein domain map of RAG1 and RAG2. **(A)** RAG1 protein domain map divided in an N-terminal non-core, core and C-terminal non-core region. **(B)** RAG2 protein domain map divided in a core and C-terminal non-core region. RAG1, Recombination-activating gene-1;ZDD, zinc dimerization domain; NBD, nonamer binding domain; DDBD, dimerization and DNA binding domain; ZBD, zinc binding domain; CTD, C-terminal domain; RAG2, Recombination-activating gene-2; PHD, plant homeodomain. Numbers indicate amino acid positions. Inspired by Schatz and Swanson (2) and Christie et al (8). Created with BioRender.com.

The *RAG1* gene is situated on chromosome 11p13 of the human genome and encodes the RAG1 protein which comprises 1.043 amino acids (aa) (6–8). The RAG1 protein can be subdivided into the N-terminal non-core region (aa 1-384), the core region (aa 384-1.008), and a short C-terminal non-core region (aa 1.008-1.040) (see **Figure 2A**) (2, 7, 8). The N-terminal non-core region contains nucleolar export and import domains, as well as a zinc dimerization and RING domain (2, 8–10). The nucleolar export and import domains regulate RAG1 protein levels as it moves in and out of the nucleus, where it exerts its function on the genome (11). The zinc dimerization domain (ZDD) comprises zinc-binding motifs in the form of zinc finger sequences that allow for homodimer formation (2, 12). The RING domain has a function in histone H3 monoubiquitylation and plays a role in V(D)J recombination activity (2, 10). Additionally, the N-terminal region is essential for full RAG1 activity with recombination enhancing domains and is shown to interact with several more proteins, including transcription and nuclear localisation factors, as well as non-homologous end-joining (NHEJ) components (2, 11, 13, 14).

The catalytic centre of RAG1 is located in the core region, which comprises the nonamer binding domain (NBD), the dimerization and DNA binding domain (DDBD), and the central domain holding the catalytic core. The central domain includes the heptamer binding region and shares a zinc binding domain (ZBD) with a C-terminal domain (CTD) within the core region (2, 7, 8). The NBD recognises and interacts with the nonamer sequence of the RSS, providing part of the specificity for RAG activity (2, 15–17). The DDBD has a role in binding DNA and provides another homodimerization domain for RAG1 (7, 8). The central domain holds motifs responsible for nicking ssDNA and recognising and interacting with the heptamer RSS (2, 8, 18, 19). The CTD functions as a dsDNA binding domain, although it does so non-specifically, relying on other domains and motifs for specificity (2, 8, 18, 20). Shared between the central domain and CTD, a ZBD is located which holds two zinc binding regions that interact with RAG2 (2, 8, 21, 22). Finally, the C-terminal non-core region, though small, has been reported to inhibit hairpin formation and modulate binding and cleavage activity together with the C terminus of RAG2 (2, 8, 23).

The *RAG2* gene is also located on chromosome 11p13, encoding the RAG2 protein comprising 527 aa (2, 7, 8, 24). Similar to RAG1, RAG2 can be divided into two distinct regions: the core region (aa 1-351) and the C-terminal non-core region (aa 352-527) (see **Figure 2B**) (2, 7, 8). The core region of RAG2 contains a six bladed β-propeller shaped by six Kelch-like motifs, which enables efficient DNA cleavage and establishes a connection with RAG1 (2, 7, 8, 21). The non-core domain comprises a hinge and a plant homeodomain (PHD) (7, 25, 26). The hinge domain provides RAG2 with a flexible connection between the core region and the PHD. This flexibility is essential for RAG’s recombination activity, as research indicates that neutralisation of the hinge region, which is relatively acidic, increases genomic instability (8, 26). Within the hinge domain reside two functional regions important in recombination: the immunoglobulin κ (Igκ) demethylation motif and autoinhibitory motif (8, 27, 28). Demethylation of the Igκ locus by RAG2 may contribute to allelic exclusion, limiting additional local recombination (8). The autoinhibition motif is regulated by binding of histone H3 on lysine 4 containing a tri-methylation (H3K4me3) (28). Relief of autoinhibition by H3K4me3 triggers DNA cleavage and recombination by the PHD in RAG2 (8, 28–30). At the C-terminal non-core region of RAG2, degradation of RAG2 is promoted by phosphorylation, and additionally by ubiquitination by Skp2 ubiquitin ligase in a cell-cycle dependent manner (2, 8, 31, 32).

In the absence of DNA, RAG1 is predominantly present in a homodimer form, while RAG2 can exist as a monomer, dimer or even unresolved larger forms (33). Together, RAG1 and RAG2 form a “Y” shaped heterotetrametric structure comprising two heterodimer arms of RAG1 and RAG2 (25, 33). RAG1 forms the base of the “Y” shape, with RAG2 located on the upper tips, and the catalytic centre situated in the middle of the “Y” joint (25, 33). For a complete detailed and in-depth view of the exact (crystal) structure of RAG we refer the reader to seminal work by Kim et al., 2015 (25).

### 2.2 Site recognition by RAG is mainly determined by chromatin features and RSSs

The recombination process begins with recognition of a suitable cleavage site. As one can imagine, the recombination process is an impactful genome editing mechanism that may cause serious problems if left unchecked. Thus, it only occurs at its intended sites, the V(D)J regions in the genome, and specifically in developing T and B lymphocytes. RAG exerts its function on the genome at sites where it detects signs of transcriptionally active chromatin and recognises the specific RSSs nonamer and heptamers (30, 34, 35). As noted above, RAG2 requires histone modification H3K4me3 in order to lift the autoinhibition of its PHD to catalyse RAGs function (28, 29). Additionally, H3K27 acetylation (H3K27ac) has been found to correlate with RAG binding to DNA (2, 8, 36). Interaction with H3K27ac was found to be RAG2 independent and seemed more dependent on N-terminal regions of RAG1, however, no clear evidence has proved this point yet (8, 36). Small sections within the V(D)J coding segments show highly active chromatin, promoting the recruitment of RAG to these sites, likely via the aforementioned histone interactions (2).

Furthermore, RAG cleavage activity is greatly dependent on nonamer and heptamer RSS recognition and binding by RAG1 NBD and the heptamer binding region in the central domain (2, 8). The NBD recognises the nonamer sequence ACAAAAACC, which binds strongly to this region with several highly conserved base pairs (bp) (8, 37, 38). The relatively weaker binding site of the heptamer CACAGTG only has the first 3 bp which are highly conserved and essential for DNA cleavage by RAG (8, 37, 38). Between the RSS nonamer and heptamer lies the spacer, which is always 12 or 23 bp long. RAG may only bind efficiently to one 12RSS and one 23RSS, not two 12RSSs or two 23RSSs. This is referred to as the 12/23 rule (1, 2, 8). It has been debated between multiple publications whether the 12RSS or the 23RSS pair is bound first or second by RAG prior to DNA cleavage (35, 39–41). Perhaps a more appealing model of 12/23 binding is one found from chromatin accessibility studies with histone modifications greatly influencing RAG binding capacities. This model suggests that the binding order of 12/23 has to do with the accessibility of the chromatin the sequence is located in (2, 35, 42). However, other factors are likely involved, such as the exact nonamer and heptamer sequences, which may vary apart from the highly conserved nucleotides. Even variations in the spacer sequence have been reported to influence RAG binding affinity (2, 8, 37, 38, 43–45).

However, not all RSS are located solely between the V(D)J segments. The so-called cryptic RSS (cRSS) are commonly present throughout the rest of the genome (8, 46). RAG’s variation in recognising RSSs can lead to different DSBs throughout the genome, causing genome instability. One example is a RAG-mediated DNA break in the *c-Myc* gene where only the 3 conserved bp (CAC) of the heptamer are enough for RAG to bind the sequence there with the heptamer binding region (8, 46). These off-target effects of RAG may lead to seriously threatening fusion genes with the risk of leukaemia’s as a result (5, 47–49).

### 2.3 Recombination from germline DNA to coding joint

**Figure 3:**
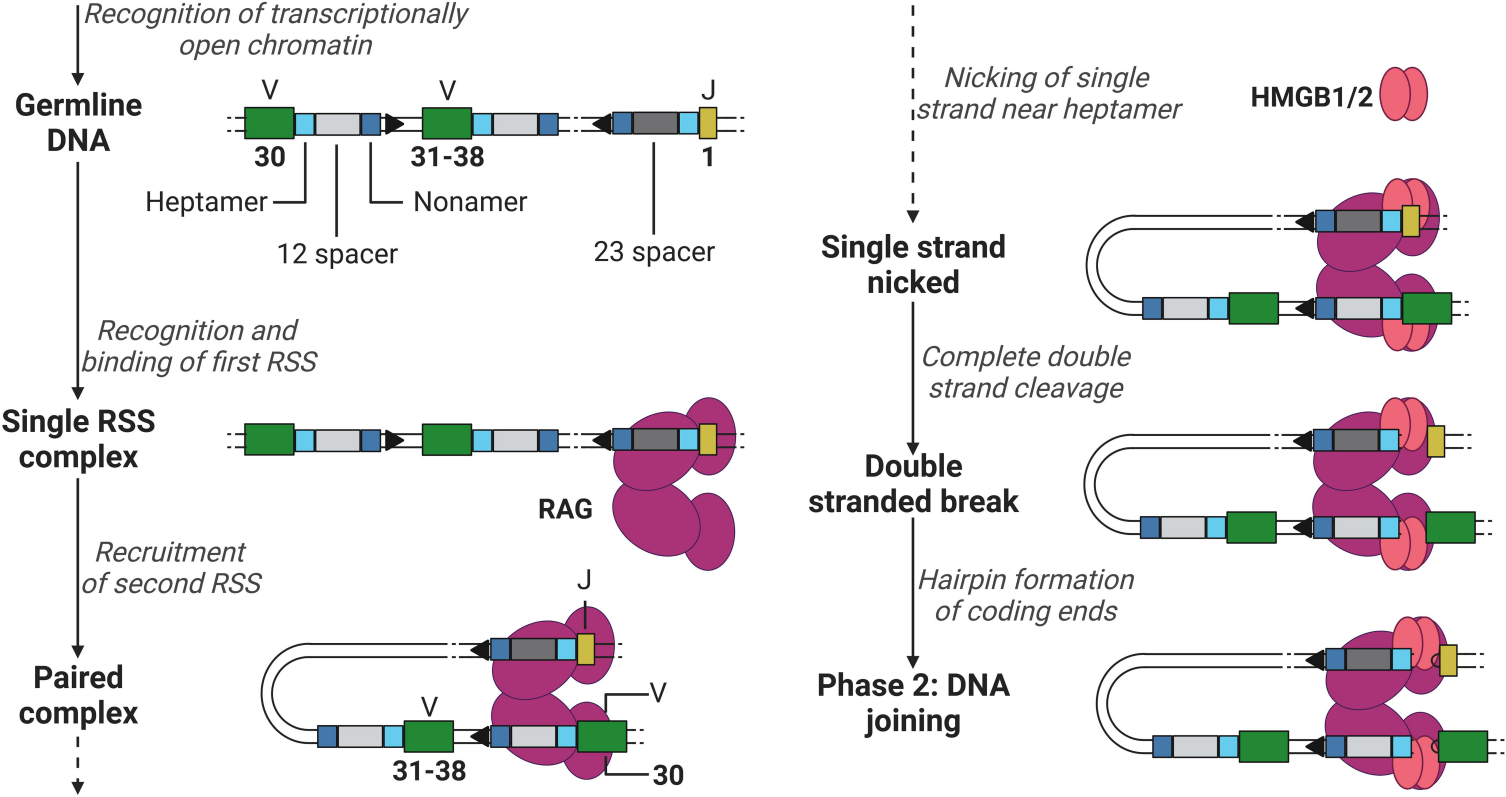
Phase 1 of recombination, DNA cleavage. Recombination example between V (green) segment 30 and J (yellow) segment 1 in a light chain, removing V segments 31 to 38. RAG (purple) binds to the first heptamer (cyan)/nonamer (blue) 23RSS (dark grey). Hereafter, binds the 12RSS (light grey) and initiates the first steps of DNA cleavage utilising HMGB1/2 (red). V, Variable; J, joining; RAG, recombination-activating gene (complex); HMGB1/2, high mobility group box 1/2. Created with BioRender.com.

The recombination process of DNA can be roughly divided into two phases, the DNA cleavage and joining phase. The first phase is initiated after RAG has gained access to the DNA via transcriptionally active histone modifications. RAG binds the first 12- or 23RSS heptamer and nonamer, forming a single RSS complex and subsequently recruits the second RSS (the 12RSS if the first was a 23RSS and *vice versa*) forming a paired complex (50). The heptamer sequences besides the 12 and 23 spacers flank the 3’ of the V segment and the 5’ of the J segment (when coding for a light chain, not containing the D segment, see example in **Figure 3**), and the nonamer sequences are positioned in between the heptamer sequences (see **Figure 3**). Once firmly in place, RAG induces a conformational change in the bound 12/23RSSs together with high mobility group box 1/2 (HMGB1/2), nicking the DNA on the 5’ single strand near the heptamer sequences, promoting an efficient double stranded cleavage initiated by RAG (see **Figure 3**) (2, 8). This cleavage creates two covalently sealed (hairpin) coding ends (at the V and J segments sites) and two blunt signal ends (at the heptamer ends).

After RAG has cleaved the DNA between the heptamer sequences and the V and J segments, the second recombination phase is initiated, the joining phase. Here, RAG dissociates from the coding ends, whilst holding on to the signal ends, giving space for NHEJ repair enzymes. Firstly, Ku70 and Ku80 heterodimers recognize the DSB site and bind to the lose DNA ends (51, 52). Once bound, Ku70/80 recruits DNA-dependent protein kinase catalytic subunit (DNA-PKcs), which forms a holoenzyme together with Ku70/80 (51, 53, 54). Initially, the Ku/DNA-PKcs complex blocks any other factor from binding to the damaged DNA site. However, DNA-PKcs in the presence of DNA damage will be trans-phosphorylated by ataxia-telangiectasia mutated (ATM) and further be auto-phosphorylated, providing access to other factors to the DSB site (51, 55, 56).

**Figure 4:**
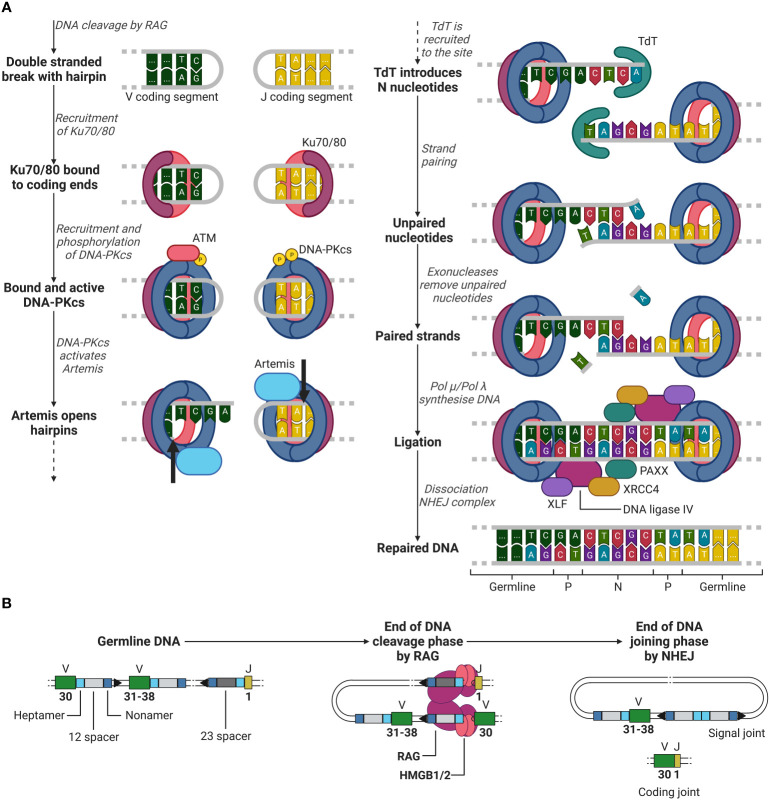
Phase 2 of recombination, DNA joining. **(A)** NHEJ example between a V (green) segment and J (yellow) segment after RAG mediated DNA cleavage. Ku70/80 (purple/red) is recruited to the double stranded break. Hereafter, DNA-PKcs (blue) is recruited, and trans/auto-phosphorylated. DNA-PKcs activates Artemis (light blue), which in turn opens the hairpins at both coding ends (cleavage location indicated by the black arrows) generating P nucleotides. TdT (cyan) then introduces N nucleotides to the P nucleotides. Strands are paired and unpaired nucleotides are removed by exonucleases. The DNA is then synthesised by either Pol µ or Pol λ and is then further ligated by DNA ligase IV (purple), supported by XRCC4 (yellow), XLF (dark purple) and PAXX (cyan). **(B)** Overview of the starting germline DNA to the end of the DNA cleavage and joining phases of recombination of a V and J segment. ATM, ataxia-telangiectasia mutated; DNA-PKcs, DNA-dependent protein kinase catalytic subunit; TdT, terminal deoxynucleotide transferase; XRCC4, X-ray repair cross-complementing protein 4; XLF, XRCC4-like factor; RAG, recombination-activating gene(complex); HMGB1/2, high mobility group box 1/2. Created with BioRender.com.

Factors such as Artemis, a 5’ to 3’ endonuclease, now have access to the DSB site and are phosphorylated by DNA-PKcs (51, 57). Artemis opens the hairpin by introducing a single strand break in the DNA behind the first to third nucleotide of the coding ends of the V and J segments (see **Figure 4A**) (51, 58). This break generates one to three palindromic nucleotides (P nucleotides) as two strands which were first complementary are now in the same strand (1, 59). Hereafter, terminal deoxynucleotide transferase (TdT), a unique enzyme only present in developing lymphoid cells, attaches two to five (on average) random nucleotides which are not originally in the germline DNA on each coding end on the P-nucleotides, generating additional diversity to the junction (1, 60, 61). These nucleotides are called N nucleotides, because they were not encoded in the germline DNA. Complementary nucleotides at the ends of the coding ends pair and unpaired nucleotides are removed by exonucleases (51). Artemis is considered to be involved in the removal of unpaired nucleotides as it possesses exonuclease activity besides endonuclease activity (51, 58). However, its role in the removal of these unpaired nucleotides is still unclear (51, 58).

Once unpaired nucleotides have been removed, DNA polymerase µ and λ (Pol µ and Pol λ) fill in the single strand DNA gaps by DNA synthesis (51). In the development of B lymphocytes, it has been shown that Pol µ is involved in the synthesis of the light chain junctions and Pol λ in the synthesis of the heavy chain junctions (61). It has yet to be investigated whether in T lymphocyte development the same distinction can be made for the use Pol µ or λ in the synthesis of the α and β (or γ and δ) TCR chain junctions. After all nucleotides are filled in, ligation is initiated by a complex of DNA ligase IV, X-ray repair cross-complementing protein 4 (XRCC4), XRCC4-like factor (XLF) and PAXX, completing the coding joint between the V and J segments (as by the example in **Figures 3**, **4**) (1, 51, 62–65). Meanwhile, the signal ends of the two cut off heptamers are repaired in a similar manner with NHEJ and form the signal joint (see **Figure 4B**) (1).

## 3 Recombination is restricted and regulated by a complex locus, various transcription factors, and cyclin dependent kinases

### 3.1 The complex locus and transcriptional regulation of the RAG genes

Multiple tiers of regulatory elements orchestrate the regulation of *RAG* gene expression (66, 67). Extensive research conducted on the *RAG* locus has unveiled several cis-regulatory elements (68). However, the mere presence of these cis-elements falls short in elucidating the strictly controlled expression pattern of the *RAG* genes during lymphocyte development (68). Various studies have indicated that BCR signalling in immature B lymphocytes represses *RAG* gene transcription by means of phosphoinositide 3-kinase (PI3K) and protein kinase B (Akt) (69–73). Diminished PI3K and Akt activity, mediated by B cell linker (BLNK) adaptor protein, leads to reduced levels of forkhead box O1 (FoxO1) and Fox3a phosphorylation (72, 73). In T lymphocytes, suppression of *RAG* gene expression has been observed to be indirectly regulated by linker for activation of T cells (LAT) and lymphocyte cytosolic protein 2 (LCP2) (74). Additionally, activation of Akt inhibits the nuclear factor of activated T-cells, cytoplasmic 1 (NFATc1), thereby exerting a negative influence on *RAG* gene expression (75).

Further investigations have demonstrated that the RAG2 promotor exhibits greater specificity for lymphoid cells compared to the RAG1 promotor (68, 75, 76). Various lymphoid-specific transcription factors, including paired box 5 (PAX5), MYB, SP1, lymphoid enhancer binding factor 1 (LEF1), nuclear transcription factor Y (NF-Y), CCAAT enhancer binding protein (C/EBP), GATA binding protein 3 (GATA3), and NFATc1, have been identified to bind to the RAG2 promotor (68, 75, 76).

Moreover, it has been established that distant regulatory elements are essential for interacting with the RAG promotors to facilitate transcription (68). These regulatory elements vary between B and T lymphocytes (68). Upstream of the *RAG2* promotor, approximately 23 kb away, lies the enhancer sequence Erag, which substantially enhances *RAG* expression in B lymphocytes (77). E2A, Foxp1, and FoxO1 are capable of binding to the Erag, thereby likely regulating enhancer activity (68, 77). Furthermore, two cis-elements situated 32 and 87 kb downstream of the RAG2 promotor have been found to govern *RAG* gene expression in T lymphocytes (78). Nestled between these cis-elements, an anti-silencing element (ASE) was found that counteracted silencing elements in the RAG locus at certain stages of T lymphocyte development (79). A common transcription factor in both B and T lymphocytes is Ikaros, which is thought to be an important regulator of *RAG* gene expression and function (80, 81).

### 3.2 Ikaros is an important regulator of RAG1 and RAG2 gene expression and protein function

An active regulator of the RAG complex is thought to be the transcription factor Ikaros (80). Ikaros has been described as a regulatory protein for TdT and is required for normal foetal T lymphocyte development, as well as in adult thymic development for pre-T lymphocyte TCR formation, and T lymphocyte differentiation choice to CD4 or CD8 (80, 82–86). Interaction of Ikaros with RAG was shown in murine B lymphocytes where hypomorphic Ikaros pro-B lymphocytes expressed lower levels of *RAG1* and *RAG2*, additionally, Ikaros knockout lymphocytes even completely lacked *RAG1* and *RAG2* expression (80, 87, 88). Later studies found that Ikaros is not only required for *RAG1* and *RAG2* expression, retroviral expression of *RAG1* and *RAG2* in Ikaros knockout B lymphocytes were unable to form functional V-DJ heavy chain rearrangements (80, 88, 89). Further studies suggests that Ikaros may be involved in light chain rearrangement in B lymphocytes as well as in allelic exclusion (80, 90–92). These studies together suggest that Ikaros is required for the expression and function of RAG genes and protein as well as allelic exclusion in the development of B lymphocytes.

### 3.3 The recombination process is restricted to the G1 phase of the cell cycle through rapid degradation towards the S phase

Multiple studies have shown that the recombination process is restricted to the G1 phase of the cell cycle in developing lymphocytes (31, 32, 93–96). RAG2 is the most studied of the two RAG proteins in this regard (97). It has been shown that RAG2 is undetectable throughout the S, G2 and M phase in dividing cells, however, during the G1 phase RAG2 accumulates and is degraded again before the S phase is initiated (32, 93). In the absence of RAG2, RAG1 aggregates in the nucleus. This aggregated form of RAG1 has an extremely low recombination activity (94). With the accumulation of RAG2 during the G1 phase, RAG1 is rescued from aggregation and forms functional RAG complexes with RAG2, allowing effective V(D)J recombination to take place (93, 94). Nearing the end of the G1 phase, degradation of RAG2 has been shown to be regulated post-transcriptionally through phosphorylation of Thr-490 by cyclin A/cyclin-dependent kinase 2 (cyclinA/Cdk2) (31, 93, 95, 96). A study further showed that *in vitro* addition of cyclin-dependent kinase inhibitor 1 (p21), an inhibitor for all cyclin-dependent kinases (CDKs) and a regulator of the G0 and 1 phase, prevented degradation of RAG2, further confirming the cyclin dependency of RAG2 (32, 98). Phosphorylation of RAG2 Thr-490 by cyclinA/Cdk2 is followed by ubiquitination by Skp2-SCF E3 ubiquitin ligase, a known regulator for G1-S transition (31, 32, 99).

Studies concerning regulation of RAG1 have demonstrated that DNA damage-binding protein 1 (DDB1) and cullin-4A (CUL4A) associated factor 1 (DCAF1) are necessary to control physiological levels of RAG1 in its protein form (97, 100, 101). RAG1 degradation is likely ubiquitin-dependent through proteasome degradation via CUL4A E3 ubiquitin ligase complexes (97). In mice, disruption of DCAF1 let to partial reduction of D-J recombination, whereas recombination of the V-DJ and V-J genes became severely impaired, resulting in a developmental block for B lymphocytes at the pro-B-to-pre-B lymphocyte stage (100). Similar to Skp2-SCF E3 ubiquitin ligase in regulation of RAG2, subunit CUL4A of the E3 ubiquitin ligase complex is a regulator of G1-S transition (102). All-in-all, these studies suggest that both RAG1 and RAG2 protein are regulated to function exclusively during the G1 phase and are marked for degradation through proteasomes during transition into the S phase of the cell cycle.

## 4 Detection methods for recombination activity and specificity

### 4.1 Cell-free DNA-RAG interaction methods for binding affinity and DNA bending kinetics

Using relatively simple assays like a DNase foot printing or electrophoretic mobility shift assay (EMSA) one can determine RAG binding to DNA or even specific (c)RSS sequences (103, 104). The DNase foot printing assay works on the principle that DNA bound by protein is protected from degradation by DNases (103). When RAG is bound to a given labelled DNA, specific sequences can be protected from DNase degradation. When these conditions are loaded onto a gel electrophoresis, the protected fragments can be observed (105). EMSA relies on a shift in molecular size when protein, in this case RAG, binds to a given DNA sequence (106). This shift in molecular size can be made visible on an electrophoresis gel, indicating an interaction (106).

However, DNase foot printing and EMSA only provide information on DNA binding of RAG. More advanced DNA-protein interaction measuring methods can be used, such as single-molecule fluorescence resonance energy transfer (smFRET) or single-molecule colocalization (smCL) assays. Besides information on specific DNA ((c)RSSs) binding, smFRET and smCL provide insight on the DNA bending kinetics of RAG (107). Using smFRET one can measure the bending of RAG target DNA by designing fluorescent donor and acceptor probes which recognise the 5’ and 3’ regions of the heptamer respectively (107). Interaction of RAG with the labelled region will induce a conformational chance in the DNA, bringing the donor and acceptor in closer proximity of each other (107). This proximity change of the donor and acceptor probes can be measured in FRET efficiency (107). With smCL RAG itself is fluorescently labelled besides a labelled sequence probe for the heptamer sequence (107). When RAG binds the labelled heptamer their colocalization and dwell time can be directly observed using total internal reflection fluorescence (TIRF) microscopy in real-time (107). All-in-all, DNase foot printing, EMSA, smFRET and smCL provide good information on DNA-RAG interaction concerning binding affinity to given DNA and DNA bending kinetics for the latter two methods.

### 4.2 *In vitro* detection of signal joints and flow cytometric analysis of lymphocyte development

The methods mentioned above are all *in vitro* cell-free methods, which may not always provide all the necessary information. Measuring RAG activity in cells adds crucial cellular context of other interacting proteins that can provide valuable additional insights. However, direct measurements of RAG in cells presents challenges. RAG is so evolutionary conserved that it is difficult to obtain antibodies that can specifically mark RAG for flow cytometry or cytometry by time flight (CyTOF). Moreover, RAG is primarily located in the nucleus, and intranuclear staining, along with surface staining using flow cytometry, can be quite challenging. An alternative approach is to use PrimeFlow™, an *in situ* hybridization assay, to demonstrate RAG gene expression in flow cytometry. However, this approach does not provide information on the RAG protein, only RNA.

Indirect effects of recombination are easier to detect, such as lymphocyte development. Using flow cytometry, one can follow the developmental stages of lymphocytes and observe hindrances or a complete arrest of development when RAG is mutated or disrupted (108, 109). Recombination activity can be indirectly measured by (q)PCR of the signal joints from *in vitro* cultures. Furthermore, cells can be transduced with an inverted reporter gene (such as green fluorescent protein (GFP)) flanked by 12- and 23RSSs, along with RAG1 and RAG2, if the cells do not express RAG endogenously (94, 110, 111). Active RAG can perform recombination at the 12- and 23RSS flanking the reporter gene, generating a readable signal from the now-active reporter gene in the signal joint (94, 110). Moreover, this method can determine the recombination efficiency of different (c)RSSs by modifying the flanking RSSs of the inverted reporter gene (94, 110).

### 4.3 Indirect methods for measuring recombination activity in an *in vivo* setting

In an *in vivo* setting, the closest approximation to a real recombination scenario is achieved. However, measuring RAG activity directly *in vivo* is very challenging and has yet to be accomplished. Indirect measuring of the recombination activity is available in the form of the TCR excision circle (TREC) assay, following lymphocyte development in the bone marrow, thymus and peripheral blood through flow cytometry, repertoire analysis, and serum Ig quantification (108). These methods can be applied, for example, on mice, where RAG-deficient mice are available and serve as good negative controls and targets for RAG gene therapy (108, 112).

### 4.4 New-born screening for early SCID diagnosis using TREC assay

Clinical outcome for SCID has been greatly enhanced with early diagnosis and treatment (113). At birth, SCID affected infants often appear in good health, as a consequence the condition is diagnosed later, often when the infant already has multiple infections and presents with secondary organ damage (113–115). In 2010 the Department of Health and Human Services (HHS) in the USA recommended screening for SCID to be included in the new-born screening, and from 2018 and forward, all new-borns are screened for SCID throughout all states of the USA (116). Since the advice of the HHS, multiple countries like Taiwan (2012), Israel (2015), Iceland, New Zealand, Norway (2017), The Netherlands (2018), Switzerland, Sweden, Germany (2019) and Denmark (2020) have implemented SCID in their new-born screening programs (117, 118).

SCID is diagnosed using the TREC assay, a simple but effective PCR of the signal joint (113, 119, 120). TRECs are the signal joints that form after recombination (see **Figure 4B**) and therefore are abundantly present in recently formed T lymphocytes. Because TRECs are not replicated during cell division, they are divided over one of two daughter cells after division, and therefore only present in a fraction of the peripheral T lymphocytes. A TREC analysis, provides quantitative information on the replication rate of these cells (119). In healthy new-borns, TRECs represent about 10% of the peripheral T lymphocytes, and they in turn reflect thymic formation of naïve T lymphocytes (119, 121).

## 5 Dysregulation of RAG is associated with cancers, autoimmunity and immunodeficiency

### 5.1 Potency of oncogenic genomic alterations in dysregulated recombination

Dysregulated recombination has been shown to cause genomic instability in lymphoid cells (5, 47–49). Some translocations, amplifications and deletions in ALL could be traced back to recombination, as (c)RSS could be detected near breakpoints in the DNA (5, 122). In comparison to other non-lymphoid malignancies like breast, prostate and pancreatic cancers, these (c)RSS were not observed near translocations, amplifications or deletions present in their representative malignant cells (5, 122). However, these genomic alterations may not be the actual cause of malignancy in lymphoid cells, but a result of previous oncogenic alterations leading to further instability of the cell through RAG dysregulation (122).

Mutations in RAG itself may alter its activity. Failure to mark RAG2 for degradation by cyclinA/Cdk2 through mutation of its substrate, Thr-490, has shown to disconnect recombination activity from the G1 phase of the cell cycle, inducing lymphoid malignancies in *p53* deficient mice (31). Furthermore, mutations in the autoinhibitory region of RAG2 may lift the histone recognition signal for activation, triggering increased off-target activity of RAG (8, 28). Besides dysregulations in RAG itself, dysregulation of NHEJ after DSB by RAG may result in genomic instability. *In vivo* murine studies have shown that RAG activity in *p53* and NHEJ deficient mice resulted in genomic instability and leukemogenesis, often presenting with aneuploidy, amplification and chromosomal translocations (123–126). However, potentially oncogenic translocations caused in mice deficient for different components of NHEJ were not equal (123). Deficiencies in *Prkdc* (DNA-PKcs) did not result in a translocation between Ig heavy chain on chromosome 12 with *c-Myc* on chromosome 15, which were recurrent in mice deficient in *p53* and *KU80, LIG4* or *XRCC4* (126, 127). All-in-all, dysregulation of RAG and/or NHEJ increases the likelihood of oncogenic genomic alterations, however dysregulation of recombination may not always be the cause for lymphoid malignancies, but a result of already preleukemic conditions in a cell (122).

### 5.2 Partial recombination deficiency may allow inclusion of self-reactive antigen receptors

Immunodeficiencies with partial recombination activity due to hypomorphic RAG mutations have been shown to carry risk for generating autoantibodies (128–131). When recombination is not completely impaired, functional lymphocytes can still develop, although restricted and not always in normal quantities (128–130, 132, 133). And just like during normal lymphocyte development, auto-reactive receptors are formed. In normal development these receptors are either edited, deleted or the cell becomes anergic (1). In the case of receptor editing, recombination is directly involved by trying other combinations of V(D)J segments if they are still possible to make (134). With partial recombination activity, this process may be impaired as well, resulting in an impaired tolerance selection if elimination or anergy fails (130). Studies have shown that partial recombination activity influences B lymphocyte tolerance in the periphery through generating a severely restricted B lymphocyte repertoire. These restricted B lymphocytes can become stimulated in an inflammatory environment which is often present in the form of infections for recombination impaired individuals, increasing the risk for autoimmunity (130, 135).

### 5.3 Majority of RAG mutations cause abnormal lymphocyte development, resulting in phenotypically heterogenous SCID

A complete or greatly deficient recombination machinery severely hampers lymphocyte development at an early stage, resulting in SCID (3, 4, 128, 136). SCID represents a heterogenous group of immunological disorders characterised by abnormalities in the function and development of T lymphocytes (and B lymphocytes, however not in all forms of SCID) (128, 137). Mutations in RAG make up a large part of SCID cases in humans (3). The most severe phenotypes of RAG deficiencies include T- and B-lymphocyte negative SCID, Omenn syndrome (OS), γδ T^+^ SCID and atypical SCID (128). Other RAG deficient immunodeficiencies with different clinical phenotypes cover: combined immunodeficiency associated with granulomas and/or autoimmunity (CID-G/AI), idiopathic CD4^+^ T cell lymphopenia, common variable immunodeficiency, IgA deficiency, selective deficiency of polysaccharide-specific antibody responses, hyper-IgM syndrome, and sterile chronic multifocal osteomyelitis (138–144). Specific mutations in RAG determine the phenotype of the disease, for a detailed list of known disease causing mutations in RAG we would refer the reader to an excellent review by Notarangelo et al., 2016 (128).

Amorphic mutations in RAG cause a complete loss-of-function in recombination, causing T and B lymphocyte negative SCID. T and B lymphocyte negative SCID can have two distinct phenotypes, one associated with a RAG deficiency, and one with a NHEJ deficiency. In the case of NHEJ deficiency, besides patients being severely immunocompromised, they also are radiosensitive due to the lack of NHEJ in DNA damage repair from ultraviolet light or ionizing radiation sources (128, 145).

OS is caused by hypomorphic mutations in RAG and still shows some recombination activity (146). OS is characterised by generalised erythroderma, lymphadenopathy, hepatosplenomegaly, eosinophilia, severe hypogammaglobulinemia with elevated levels of IgE, and multiple organs infiltrated with activated T lymphocytes (128, 147, 148). It is speculated that T lymphocytes in these patients are skewed towards T helper 2 lymphocytes, causing elevated levels of serum interleukin-5, it is however unclear how this skewing is initiated (128). Furthermore, distinct restrictions in TCRs may induce autoimmunity, causing T lymphocyte infiltrations in multiple organs (128, 148, 149). One explanation may be that specific mutations may alter V(D)J recombination in a particular way, favouring certain RSS above others (20, 128). In some conditions of autologous T lymphocytes without typical OS phenotype, the disease is referred to as atypical SCID (3, 150–152).

Some cases of hypomorphic RAG show relatively normal numbers of γδ T lymphocytes despite the patients having αβ T lymphocyte lymphopenia (153, 154). This so called γδ T^+^ SCID phenotype has been associated with cytomegalovirus (CMV) infection and does not present itself with the characteristic features of OS (128, 153, 154). It has been suggested that the expansion of γδ T lymphocytes is antigen-driven by the CMV infection, causing peripheral clonal expansion of these cells (153, 154). Patients with γδ T^+^ SCID phenotype often develop autoimmune reactions despite having minimal levels of B lymphocytes, however they do respond to some vaccines and infections (154). All-in-all RAG deficiencies can present themself in many different phenotypes depending on the nature of the mutation in RAG or NHEJ components, or the environmental exposure to pathogens, resulting in life-long and life threatening immunodeficiencies, often combined with autoimmunity (128).

## 6 Gene therapy for RAG-SCID

### 6.1 Gene therapy-based precision medicine as most suitable therapeutic intervention for RAG deficient SCID

For SCID, the curative treatment exists in the form of haematopoietic stem cell (HSC) transplantation, however, a well matched donor is not always available. Proper HLA matching decreases the chances of Graft versus Host Disease (GvHD) as mismatched HLA in an allograft transplant often results in Graft versus Host Disease and other comorbidities (155). An autologous bone marrow transplant with corrected genes, also known as autologous gene therapy, would be the ideal cure for these diseases. *Ex vivo* gene therapy treatment allows for control and selection before transplanting the treated cells back to the patient. This form of gene therapy may be achieved in different ways, through retro- or lentiviral vector transduction with corrected genes (gene addition) or by gene editing in which the affected locus is corrected through the use of nucleases, nowadays most frequently Clustered regularly interspaced short palindromic repeats (CRISPR)/Cas.

Upon infection of a host cell, retroviruses integrate their genetic information more or less permanently in the host genome (156). Retroviruses have thus been seen as a natural gene delivery system and were actually used in the first recorded case of successful human gene therapy in the 1990s (157, 158). Besides a few of the viral DNA domains and long terminal repeat (LTR) elements, a large part of the viral genetic information can be replaced by exogenous DNA, up to 8 Kbp (159). The virus can be further modified by pseudotyping, replacing the envelope gene encoding for envelope glycoproteins with envelope genes of other viruses, changing the infectable host cells (159). Upon infection, integration is only possible during cell division, one of the downsides of a gamma-retroviral vector. Due to their specific integration proteins, integration by gamma-retroviral vectors is most likely to take place in euchromatin rather than heterochromatin, risking insertional mutagenesis which has led to cases of acute leukaemia in gamma-retroviral vector treated patients (159–165). However, since the first use of gamma-retroviral vectors, safety has been improved with insulator sequences that modulate promotor activity and the development of self-inactivating (SIN) vectors (166–169).

Close relatives to the gamma-retroviruses are lentiviruses and they too can be considered a good tool for gene delivery in gene therapy (and other applications) (159). Lentiviruses are considered to be more complex than gamma-retroviruses and thus are more complex to modify for specific gene delivery strategies. However, they come with more advantages as well. Lentiviruses come with additional regulatory genes that allow for integration in non-dividing cells (170, 171). Appropriate pseudotyping and modification of lentiviruses has resulted in space for up to 9 Kbp of exogenous DNA and the ability to effectively target and transduce difficult-to-transduce cells such as HCS, lymphoid cells, some myeloid cells, neurons and others (159, 172–175). Additionally, lentiviruses have low LTRs inducible promotor activity due to SIN modifications, and tend to integrate further away from start sites of cellular promotors, lowering the risk of insertional mutagenesis and oncogenicity compared to gamma-retroviruses (159, 160, 176).

In the ideal situation however, one should actually repair the mutated gene instead of inserting an additional one. Gene editing by CRISPR has great promise in this regard. Using CRISPER/Cas9, a DSB can be introduced in the mutated gene with a single guide RNA (sgRNA). This break can then be repaired through the cells homology-directed repair (HDR) mechanisms using donor DNA with homologous arms to the DSB site. This mechanism of gene editing can transport up to 4,7 Kbp of exogenous DNA and can be delivered via adeno-associated virus (AAV), which can be modified to target HSCs effectively (177–181). Because this method of gene editing relies on HDR, it can only be executed in dividing cells (182). Furthermore, CRISPR/Cas9 is not error-free, although great advances are being made to eliminate off-target activity (183–186). However, some studies using *ex vivo* gene editing with CRISPR/Cas9 transported in AAV with a donor sequence, triggered immune responses, negatively influencing the stemness of HSCs and reducing their long-term seeding after transplantation (178, 179, 185, 187–189). In addition, CRISPR also shows on target side effects, due to its imprecise nature and can generate indels at distances up to several hundred bps around the desired site, possibly disrupting normal gene regulation (190, 191). Especially for a locus that is subject to strict negative control, such as the RAG locus, these safety considerations are of utmost importance.

Besides gene editing through HDR, a modified Cas9 has been engineered with base editing enzymes, reverse transcriptase’s and prime editing enzymes (177, 192, 193). These CRISPR/Cas9 fusion mechanisms can edit single nucleotides, add insertions up to 44 bp, and delete up to 80 bp at 5 to 50 bp away from the target site (193). All-in-all, CRISPR-based gene editing caries great potential for gene therapy applications, however, further improvements in safety regarding off-target editing and delivery of the machinery need to be made in order for these methods to be safely implemented in clinical gene therapy.

### 6.2 Ongoing lentiviral-based clinical trial for RAG1-SCID shows great promise in immune reconstruction

As introduced above, SCID is a prime candidate to be treated by autologous stem cell based gene therapy. Indeed, the first successful gene therapy efforts were done with retroviral vectors for treating X-linked-SCID which is caused by mutations in the *IL2RG* gene (191). Based on the initial success, a similar approach was proposed to treat RAG1-SCID. While this kind of RAG1 gene therapy showed good efficacy in the mouse models, the occurrence of leukaemia due to insertional mutagenesis, forced the field to move to SIN LV vectors (164, 194). For some types of SCID, these vectors became quickly available, however for RAG1-SCID this proved to be a formidable challenge due to the high expression of RAG1 in very strictly defined lymphoid progenitor populations (195, 196). We first reported successful LV preclinical work with the spleen focus-forming virus (SFFV) promotor, which was later replaced by the more clinically acceptable myeloproliferative sarcoma virus enhancer, negative control region deleted, dl587rev primer-binding site substituted (MND) promotor, which has been used before in clinical trials for gene therapy (109, 112, 197). Since then, preclinical studies on lentiviral-based gene therapy for RAG1-SCID has advanced to a phase I/II clinical trial (NCT04797260) in 2021 (108, 109, 112, 198). These preclinical murine studies used RAG1 deficient mice and performed gene therapy with a lentiviral SIN vector containing codon optimised *RAG1* (*coRAG1*). The treatment showed successful reconstruction of T and B lymphocytes in peripheral blood and developing lymphocytes seeded central lymphoid organs (109). And when challenged with foreign antigens, treated mice showed an antigen-specific immune response (109). Importantly, a relatively high level of *coRAG1* expression was essential for successful recombination, as low levels of *coRAG1* due to inefficient transduction resulted in OS-like T lymphocyte phenotypes (198, 199). After passing regulation standards, the lentiviral *coRAG1* vector moved on to a phase I/II clinical trial, and as the clinical trial progresses, we eagerly await the results for the human *in vivo* setting. Thus far, two patients have been included and a third one will be transplanted with corrected autologous stem cells soon.

Recent work using HSC based gene therapy for cerebral adreno- leukodystrophy (CALD) using a similar MND promoter has showed 3 cases of myelodysplastic syndrome out of 70 patients treated showing the risk of insertional mutagenesis that is associated with this kind of therapy (197). For RAG1 LV gene therapy we tested 7 different promoters as high RAG1 expression is required to restore T cell development (108, 112, 198, 200). In safety assays, the MND-coRAG1 vector has not shown signs of insertional mutagenesis (112, 200). We have also opted for transduction efficiencies with approximately one integration per target cell, instead of VCNs of 5 to 10 that were aimed for in the CALD trial, where indeed 3-5 viral integration sites per dysplastic clone were found (197). Further follow up will show whether the perceived lower change of xeno-toxicity events in the RAG1 SCID trial holds up.

For RAG2-SCID, we have developed SIN LV vectors that correct RAG2-deficiency in mouse models (200). The vector is currently being produced good manufacturing practices (GMP) grade to facilitate clean room testing leading up to a phase I/II clinical trial anticipated early 2024 (an overview of other current developments in RAG gene therapy is provided in **Table 1**).

**Table 1:** Overview of the current developments on RAG gene therapy.

RAG1/2-SCID	Gene therapy strategy	Vector/endonuclease	Stage
RAG1	Gene addition	MLV/RV	*In vivo* mice (194)
RAG1	Gene addition	LV	*In vivo* mice (109)
RAG1	Gene addition	LV	*In vivo* mice (199)
RAG1	Gene addition	LV	Phase I/II (108, 112)
RAG2	Gene addition	MLV/RV	*In vivo* mice (201)
RAG2	Gene addition	LV	*In vivo* mice (202)
RAG2	Gene addition	LV	*In vivo* mice (203)
RAG2	Gene editing	CRISPR-Cas9/rAAV6	*In vitro* iPSCs (204)
RAG2	Gene editing	CRISPR-Cas9/rAAV6	*In vitro* HSPCs (205)
RAG2	Gene editing	CRISPR-Cas9/AAV6	*In vitro* HSPCs (206)

MLV, Moloney leukemia virus; RV, retro virus; LV, lentivirus; rAAV6, recombinant adeno-associated virus serotype 6; HSPCs, hematopoietic stem and progenitor cells; iPSCs, induced pluripotent stem cells.

## 7 Conclusion

In summary, RAG plays a crucial role in the intricate process of recombination and lymphocyte development, leading to the generation of immune diversity. Thanks to extensive research, we have gained a more comprehensive understanding of RAG’s functional domains and motifs, and how it initiates the recombination process (2, 8). Furthermore, investigations into RAG have uncovered regulatory links to chromatin features, RSS, transcription factor Ikaros, the G1 phase of the cell cycle, and developing lymphocytes (2, 8, 28, 31, 80). Dysregulation of these components has been linked to diseases like cancer, autoimmunity, and SCID (2, 8, 128). However, with the emergence of precision medicine in the form of gene therapy, the possibility of treating diseases like SCID is now becoming a reality (108, 112). The current innovative methods for detecting and measuring recombination activity by RAG hold promise for advancing research on RAG deficiencies, gene therapy and related fields. However, the development of direct intranuclear measuring of RAG protein would be a significant addition to these methods, enabling even more precise and accurate measurements of recombination activity. Furthermore, the continued development of error-free CRISPR gene editing, efficient transduction delivery systems, and safe viral vectors will further enhance the potency of gene therapy, providing hope for patients suffering from a wide range of genetic disorders. As these technologies continue to evolve, it is likely that the future of gene therapy will be characterised by even greater precision and efficacy, paving the way for a new era of personalised medicine.

The first gene therapy trial for RAG1-SCID (NCT04797260) is currently underway, and the outcome is eagerly anticipated (108, 112). With existing literature providing strong support for further development of gene therapies for forms of SCID and other genetic disorders, advanced culture systems such as artificial thymic organoids (ATOs) can further facilitate this research by allowing for the use of patient material in culture and reducing the need for animal experimentation. However, creating effective and safe gene therapy for SCID poses significant challenges, given the powerful genome editing tools involved. As such, meticulous regulation is necessary to ensure the successful implementation of gene therapy for RAG in SCID. By carefully balancing innovation with regulatory oversight, we can continue to make strides towards safe and effective gene therapies that have the potential to transform the lives of patients with genetic disorders.

## Author contributions

All authors listed have made a substantial, direct, and intellectual contribution to the work and approved it for publication.
